# The relationship of multiple sclerosis and cerebral developmental venous anomaly with an advantageous role in the multiple sclerosis diagnosis

**Published:** 2017-10-07

**Authors:** Mohammad Reza Sasani, Ali Reza Dehghan, Nikseresht Ali Reza

**Affiliations:** 1 Medical Imaging Research Center, Shiraz University of Medical Sciences, Shiraz, Iran; 2 Department of Radiology, School of Medicine, Shiraz University of Medical Sciences, Shiraz, Iran; 3 Autoimmune Diseases Research Center, Shiraz University of Medical Sciences, Shiraz, Iran; 4 Department of Neurology, School of Medicine, Shiraz University of Medical Sciences, Shiraz, Iran

**Keywords:** Multiple Sclerosis, Cerebral Venous Angioma, Pathogenesis, Intracranial Central Nervous System Disorders, Venous Insufficiency, Magnetic Resonance Imaging

## Abstract

**Background:** There is a suggestion for a role of abnormal cranial venous drainage in the etiopathogenesis of multiple sclerosis (MS). Moreover, it seems that cerebral developmental venous anomaly (DVA), a cerebrovascular malformation, is frequently seen in the magnetic resonance imaging (MRI) of MS patients. This study is set out to evaluate the relationship between MS and cerebral DVA, with its possible role in the MS diagnosis.

**Methods:** We compared MRI of 172 MS patients and of 172 age- and sex-matched subjects without MS. Then, we recorded and analyzed the presence, number, and location of developmental venous anomalies.

**Results:** Frequency of DVA did not have a significant statistical difference (P = 0.148) in subjects with MS (12.21%) and without MS (7.55%). Moreover, a difference of anatomic distribution of supratentorial developmental venous anomalies was not statistically significant (P = 0.690, for juxtacortical, P = 0.510 for subcortical, and P = 0.420 for periventricular DVAs) in two groups.

**Conclusion:** Our investigation does not provide supporting evidence for a relationship between etiopathogenesis of MS and DVA. Furthermore, it may not be possible to use cerebral DVA as ancillary MRI finding to make MS diagnosis simpler and more accurate.

## Introduction

Multiple sclerosis (MS) is an inflammatory demyelinating disease of central nervous system,^1^^,^^2^ identified from more than one century,^3^ but its etiology is still in doubt.^4^ Recently, abnormal cranial venous drainage has been described to have a possible role in the etiopathogenesis of MS. On the other hand, it has been suggested that the area of brain affected by developmental venous anomaly (DVA), also known as venous angioma, does not have normal venous drainage.^5^ Additionally, it seems that cerebral DVAs are frequently seen in the brain magnetic resonance imaging (MRI) of MS patients. These explanations raise the question whether MS and DVA have any association. Furthermore, there is an issue about the coincidence of MS and DVA as well as an advantageous role of DVA (as an ancillary finding in brain MRI) in the diagnosis of MS.

Histopathologically, there is perivenular inflammation and demyelination in MS plaques,^6^^,^^7^ which are located characteristically in juxtacortical, periventricular, or infratentorial regions of the brain.^8^ Moreover, categorization of DVAs into three groups of juxtacortical, subcortical, periventricular is relevant to their location and drainage pattern.^9^ In fact, these explanations may support the relationship of MS and DVA.

Another area for consideration is the era of MS diagnosis. MRI is salient imaging modality for diagnosing MS with the use of McDonald criteria.^1^^,^^10^ Although MRI is a sensitive tool to find MS plaques, its specificity is low and might lead to a fault in the diagnosis especially in the early period of MS.^1^^,^^7^^,^^10^ The reason is that the other conditions such as a migraine and microvascular disease, which have hypersignal white matter foci, could simulate MS plaques.^7^ Consequently, the recognition of ancillary findings in brain MRI might help to diagnose MS simpler and more accurately. During our daily work, the DVAs have been frequently encountered in brain MRI of patients with lesions suspicious to MS. It has been proposed the idea that one of the ancillary findings may be the presence of cerebral DVA. We believe that it is essential to conduct a study to address questions about the possible relationship and the coincidence of MS and DVA. 

Although several studies have been carried out to evaluate the possible relationship between abnormal cerebral venous flow and MS, there is a controversy about this topic. Furthermore, it has been suggested that the coincidence of vascular malformation and MS might happen but little attention has been drawn to this topic in the literature.^11^ A research, using transcranial color duplex sonography, concluded that altered venous flow could have a possible role in MS inflammatory process.^12^ Another multicenter study with use of color Doppler sonography reported that there is a relationship between chronic cerebrospinal venous insufficiency and MS.^13^ However, they have suggested using more reliable imaging modality due to variable data about the diagnosis of chronic cerebrospinal venous insufficiency in different centers.^13^ By contrast, another study utilizing magnetic resonance venography (MRV) and MR flow quantification showed equal distribution of anomalous cranial venous outflow in MS patients and healthy subjects.^14^ They finally concluded that mentioned anomalous cranial venous outflow probably stand for anatomical variations of venous drainage rather than abnormalities that might have relationship with MS.^14^

The purpose of this study is to compare the frequency and anatomical distribution of cerebral DVA in MS patients and healthy subjects in order to emphasize on the relationship of their etiopathogenesis along with identification of DVA as an ancillary MR finding. It may help to make MS diagnosis simpler and more accurate.

## Materials and Methods

This case-control study was conducted to compare the frequency and anatomic distribution of DVAs in patients with MS and subjects without MS. A total of 344 participants were recruited for this study. Eligible cases consisted of 172 patients with definite diagnosis of MS were referred from neurology clinic. Control group consisted of 172 subjects without MS were referred to perform MRI with non-specific reason. Subject demographics are presented in table 1. The mean age ± standard deviation (SD) of the case group was 32.75 ± 0.57 years, while it was 32.44 ± 0.58 years for the control group (age range in both groups was 18-50 years). Case and control groups were matched by age and sex. The local ethical committee approved this study and written informed consent was obtained from the participants.

**Table 1:** Sex and age distribution in case and control groups

**Group**	**Man [n (%)]**	**Woman [n (%)]**	**Age (year) (mean ± SD) **	**Age range (year)**
Case (n = 172)	25 (14.5)	147 (85.5)	32.75 ± 0.57	18-50
Control (n = 172)	25 (14.5)	147 (85.5)	32.44 ± 0.58	18-50

Exclusion criteria for case group were the presence of pathologies other than MS, such as malignancy, meningoencephalitis, vasculitis, hematopoietic disorders and history of other immunological diseases. Exclusion criteria for the control group were suspicious MS (clinically or radiologically); the presence of any abnormality (except for DVA) in MRI, or previous history of malignancy, meningoencephalitis, vasculitis, hematopoietic disorders, and other immunological diseases.

Brain MRI images were acquired using MRI systems operating with a magnetic field strength of 1.5 Tesla (Magnetom Avanto mobile MRI 02.05, software version: Syngo MR B15; Siemens Ltd, Erlangen, Germany) and the following sequences were obtained: Axial and coronal T2-weighted sequences, axial FLAIR (fluid attenuated inversion recovery) sequence, sagittal proton density-weighted sequence, axial and sagittal pre-contrast T1-weighted sequences as well as axial, sagittal and coronal post contrast T1-weighted sequences after administration of 0.1 mmol/kg of gadolinium contrast agents including Magnevist (gadopentetate dimeglumine, Germany) or Omniscan (gadodiamide, Ireland) or Dotarem (gadoteric acid, France). 

All the MRI images studied by one radiologist and the presence, number, and location of DVAs were recorded. The diagnosis of DVA was based on its appearance on MRI images as multiple enlarged enhancing vessels with star-like configuration draining into a collecting vessel. The DVAs were assorted as supratentorial and infratentorial. In addition, supratentorial DVAs were categorized into three subgroups as juxtacortical (within a gray matter or at the junction of gray and white matter), periventricular (adjacent to ventricles), subcortical (between the juxtacortical and periventricular area).

Data analysis was performed using SPSS software (version 17, SPSS Inc., Chicago, IL, USA). The case and control groups were compared to each other by using the chi-square test and different variables were correlated with Pearson correlation. P less than 0.050 were regarded as statistically significant.

## Results

A total of 344 participants (172 cases and 172 controls) were recruited for this study. Case and control subjects had similar age and sex distribution without significant difference (P = 0.070). Twenty-one (12.21%) patients of the case group and 13 (7.55%) subjects of the control group had DVAs. There was no significant difference between the two groups regarding frequency of DVAs (P = 0.148). 

**Figure 1:**
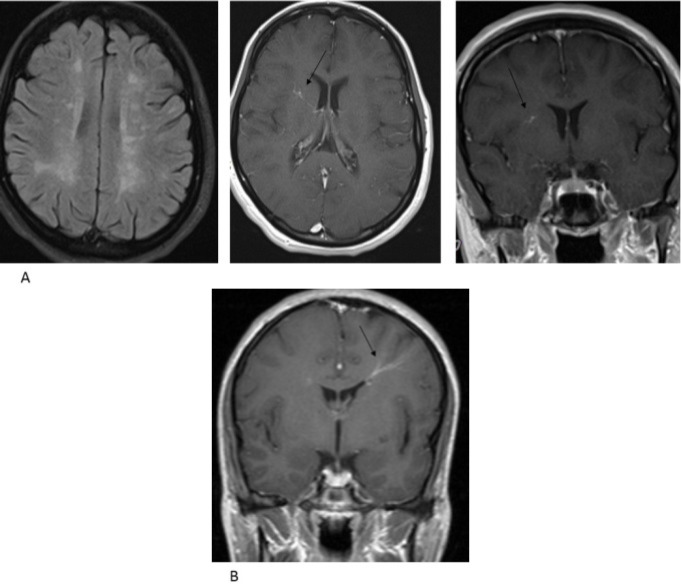
Developmental venous anomaly (DVA) in a patient with multiple sclerosis (MS). Axial fluid-attenuated inversion recovery image shows MS (multiple sclerosis) plaques and post-contrast T1-weighted images show enhancing vessels joining the collecting vein (arrows) (A), Developmental venous anomaly (DVA) in a control subject. Post-contrast T1-weighted image shows enhancing vessels joining the collecting vein (arrow) (B).

Of the 21 DVAs in the cases, 18 were supratentorial (Figure 1) and three were infratentorial. In the control group, three subjects had more than one DVA and a total of 17 DVAs (16 supratentorial, 1 infratentorial) detected in this group. The analysis did not reveal a significant difference between two groups on the subject of supratentorial DVAs (P = 0.400). Because of a limited number of infratentorial DVAs, they were not analyzed.

Table 2 summarizes the anatomical distribution of supratentorial DVAs. The difference between cases and controls on the subject of juxtacortical, subcortical, and periventricular DVAs was tested. P for juxtacortical DVAs was 0.690, for subcortical ones was 0.510, and for periventricular ones was 0.420. None of these differences was statistically significant.

## Discussion

In this study, the frequency of DVA in patients with MS was 12.21% and in subjects without MS was 7.55%, in which their difference was not significant statistically. Moreover, there was no statistically significant difference between cases and controls in terms of anatomic distribution of DVAs. We were surprised to find higher value for DVA frequency in subjects without MS, approximately 7.55%, as compared to previously reported frequency of DVA in the literature, which had been less than 2%.^5^^,^^15^^,^^16^

**Table 2:** Anatomical distribution of supratentorial developmental venous anomaly (DVA) in case and control groups

**Supratentorial DVA**	**Case ** **[n (%)]**	**Control ** **[n (%)]**	**P** *
Juxtacortical	3 (16.6)	3 (18.8)	0.690
Subcortical	7 (38.9)	8 (50.0)	0.510
Periventricular	8 (44.5)	5 (31.2)	0.420

DVA: Developmental venous anomaly

* Chi-square test

This investigation does not provide additional support for the association between anomalous cranial venous drainage and MS. Although the frequency of DVA in cases and controls was different, it was not statistically significant indicating that DVA is not more common in MS patients. Accordingly, it may not be possible to utilize cerebral DVA as ancillary MR finding for the diagnosis of MS. Moreover, anatomical distribution of DVA in the brain shows no significant correlation with the characteristic location of MS plaques. In fact, our results do not reinforce the association of MS and DVA, as an example of anomalous venous drainage.

Our results are consistent with previous works that claimed there is no relationship between chronic cerebrospinal venous insufficiency and etiopathogenesis of MS. In a study, using phase contrast MRI and with focus on the internal jugular vein, there was no supporting evidence for vascular MS hypothesis.^17^ In another study, no association between chronic cerebrospinal venous insufficiency and lesion burden in MS patients could be found.^18^ Results of another investigation were against the significant role of venous congestion in MS pathogenesis.^19^

As noted, this study shows a higher value for the frequency of DVA in subjects without MS. This finding can be justified in part by our study population, which control subjects were not selected from the general population. Another possible explanation for this may be that some DVA cases were not diagnosed in the past years due to the lower magnetic field strength of MRI systems or lower quality of their images. Therefore, we recommend that further research should be undertaken in this area.

A limitation of our research is that controls were not selected from the general population and they consisted of subjects without MS who referred to perform MRI with non-specific reason. However, we excluded subjects with suspicious MS (clinically or radiologically), those with any abnormality in MRI, and those with positive past history (as previously mentioned).

## Conclusion

Frequency and anatomical distribution of cerebral DVA in patients with MS do not reveal a significant difference in comparison with subjects without MS. Consequently, our investigation does not provide supporting evidence for the relationship of the etiopathogenesis of MS and DVA, as an example of anomalous venous drainage. Furthermore, it may not be possible to utilize cerebral DVA as ancillary MR finding to make MS diagnosis simpler and more accurate.

## Notes:

**How to cite this article:** Sasani MR, Dehghan AR, Nikseresht AR. The relationship of multiple sclerosis and cerebral developmental venous anomaly with an advantageous role in the multiple sclerosis diagnosis. Iran J Neurol 2017; 16(4): 168-72.
